# Lottery, luck, or legacy. A review of “The Genetic Lottery: Why DNA matters for social equality”

**DOI:** 10.1111/evo.14449

**Published:** 2022-03-11

**Authors:** Graham Coop, Molly Przeworski

**Affiliations:** ^1^ Center for Population Biology and Department of Evolution and Ecology University of California, Davis Davis California USA; ^2^ Department of Biological Sciences and Department of Systems Biology Columbia University New York USA

## Abstract

A book review of “*The genetic lottery: why DNA matters for social equality*.” (Princeton University Press, 2021) by Kathryn Paige Harden.

*The Genetic Lottery: Why DNA Matters for Social Equality* aims to convince the reader that recent methodological developments in human genetics should change the broader societal conversation about redistributive justice. The author, Dr. Kathryn Paige Harden, is a Professor of Psychology at the University of Texas, Austin, who specializes in behavioral genetics. Her book starts from the premise that human behaviors, and in particular educational attainment, are “heritable,” i.e., that within a study sample, some fraction of the phenotypic variance is explained by differences in genotypes. As is described, we can now identify some of the genetic loci associated with trait variation through genome‐wide association studies (GWAS) and make predictions—currently, quite noisy predictions—of individual outcomes from genotypes. In the author's view, GWAS findings underscore that people differ not only in the social circumstances into which they are born but also in the genetics that they happen to inherit. Since neither social circumstances nor genetics are earned or chosen, both result from “luck.” The book argues that both sources of luck contribute commensurately to social inequalities in educational attainment and ultimately in income, and therefore that genetics is needed in order to better understand and redress social inequalities. In particular, in Harden's view, recent GWAS findings should lead us to be mindful of principles of equity and not just equality.

The author is an extremely talented communicator, and *The Genetic Lottery* includes discussion of many engaging and thought‐provoking examples. But in our view, its central argument mischaracterizes where the field of human genetics stands and what it promises. Although some of the controversy over the book has centered on its premise, the fact that educational attainment is heritable was documented before GWAS and is in some sense trivial. In humans as in any other species, almost all traits that vary within a group are heritable (Barton & Keightley, 2002; Turkheimer, 2000). We thus fully grant the book's starting point. We also happen to support redistributive policies outlined in her conclusions. However, we believe that many of the arguments made to connect the premise to these conclusions are unwarranted, notably concerning the pertinence of GWAS findings.

Given its broad scope, *The Genetic Lottery* presents many angles from which to comment. As others have pointed out, it focuses attention on “genetic luck,” when people face social and historical inequities that are anything but random (Martschenko, 2021), and considers the impacts of relatively small social interventions rather than the larger structural inequities in which they are embedded (Panofsky, 2021; Parens, 2021). As population geneticists, and given the importance placed on GWAS and trait prediction in the book, we concentrate on points at which the scientific results are distorted or exaggerated. Cumulatively, these mischaracterizations foster a view of genetic causes of educational attainment as identifiable, intrinsic properties of individuals. As we discuss, this view is not justified by current understanding.

## THE BACKDROP

*The Genetic Lottery* relies on findings from a number of different approaches, such as twin studies, the meanings of which have been discussed for decades (Downes & Turkheimer, 2021; Feldman & Lewontin, 1975; Lewontin, 1974; Tabery, 2008). Notably, it contends that heritability estimates of educational attainment are reflective of the extent to which variation in the trait is *caused* by genetic differences in a given setting, despite long‐standing arguments to the contrary (Lewontin, 1974; Morrissey et al., 2010; Visscher et al., 2008). What is scientifically novel about the book's argument, and the author argues, disruptive, is the evidence from GWAS.

Over the past two decades, GWAS have been performed for thousands of traits, almost always in individuals of European ancestry living in the United States or Europe, and with a bias in enrollment toward relatively wealthier people. Recorded information about participants often includes their educational attainment, usually as a categorical variable that describes the stage of schooling completed by the individual. Like almost any trait, educational attainment is heritable: the proportion of the trait variance attributed to the (additive) genetic variance varies from 17% to over 40%, depending on the assumptions of the heritability estimator and the study sample (Branigan et al., 2013; Kemper et al., 2021; Young et al., 2018). Collating information from individual GWAS has therefore permitted massive meta‐GWAS of educational attainment, most recently in 1.1 million people (Lee et al., 2018).

These GWAS are conducted in “unrelated” individuals, i.e., sets of individuals that are not close relatives. The trait value (educational attainment) is regressed on each genetic variant, with a statistical control for effects of population stratification (Price et al., 2010). Although these controls are imperfect, they suffice for the top associations to be highly reproducible across GWAS samples of similar ancestry (Lee et al., 2018). These GWAS reveal that, like most human traits studied to date, educational attainment is massively polygenic: None of the associations explains much of the variance of educational attainment, and most explain a tiny proportion. Moreover, because of correlations among alleles at nearby sites (linkage disequilibrium), the precise identity of the causal loci is often unclear. Nonetheless, given adequate control for population structure, the study design indicates that one or more variants in that general genomic location influence the trait value. How they exert that influence is almost always unknown. What is understood, however, and Harden repeatedly clarifies, is that whatever the underlying mechanism may be, behaviors such as educational attainment are the outcome of individual tendencies *as they are manifested in specific social environments*.

Despite the GWAS findings lacking a mechanistic interpretation, they can still be used for trait prediction in similar settings. Following the approach taken by quantitative geneticists for decades (Wray et al., 2019), a “polygenic score” (PGS) can be calculated for an individual by summing all or a subset of variants in their genome, weighted by the effect sizes on the trait estimated in the GWAS. These polygenic scores provide prediction of individual deviations from the mean trait value in individuals similar to the GWAS set. As Harden is careful to point out, they are statistically significant but noisy predictors of an inherently probabilistic outcome. Currently, they account for 11–13% of the variance in educational attainment in people of European genetic ancestry, depending on the cohort (Lee et al., 2018).

Polygenic scores are of interest for a number of distinct purposes, many of which figure prominently in the book. The first is as an instrument or control variable in the social sciences: for instance, using PGS (e.g., for Body Mass Index, BMI) as a covariate allows one to study the impact of an environmental intervention (e.g., an extra year of schooling) on an outcome (e.g., BMI), while statistically controlling for heterogeneity in genetic effects on the outcome (e.g.,  Barcellos et al., 2018). Uses of PGS as statistical tools are in their infancy and, although not without assumptions, seem promising. Where the book touches a nerve, we suspect, is the other applications: where it claims that PGS for educational attainment are useful predictors of scholastic achievement, and GWAS an important tool for understanding the causes of social inequalities. Here, we highlight three central issues that we believe call into question these proposed applications, and thus much of the argument of the book: (i) the elision of different types of GWAS; (ii) the interpretation of GWAS hits for educational attainment as “built in” differences among individuals; and (iii) the reliance on typological notions of populations.

## THE ELISION OF DIFFERENT TYPES OF GWAS

Rawls famously posited that a fair society is one that people would choose if they did not know the circumstances of their birth–what tickets they had drawn from social and natural lotteries (Rawls, 1971). Harden invokes the image of lottery in reference to Rawls, but also to mean something distinct and much more specific. In claiming that *“a lottery is a perfect metaphor for describing genetic inheritance”* [p. 17], she writes *“The fact that you have your specific DNA sequence out of all the possible DNA sequences that could have resulted from the union of your mother and your father, is pure luck. That is what I mean when I say your genotype […] is an outcome of a genetic lottery”* [p. 31]. This use of a genetic lottery as a form of randomization is central to the book and critical to its argument that genetics helps to identify causes of inequality.

Yet this particular usage of the term does not apply to comparisons among unrelated people, only to the transmission of alleles from parent to child or the sharing of alleles among siblings. And the effects of these alleles can only be isolated from other factors in one, relatively uncommon, type of GWAS, known as a family design. Family GWAS come in various flavors, the most common of which is a sib‐study, in which differences in trait values between biological full siblings are regressed on the differences in their genotypes. Other family designs use related approaches, distinguishing between the effects of transmitted and untransmitted parental alleles for instance (Kong et al., 2018). Their design implicitly controls for the parental environment, be it environmental or genetic, and (under some assumptions) randomizes genotypes across the environment of the children. Thus, any differences in outcomes between siblings reflect the alleles that the parents happened to transmit to their offspring, i.e., the lottery of Mendelian inheritance.

As Harden notes, this type of lottery is analogous to a randomized controlled trial, in which siblings are randomly assigned different treatments (i.e., PGS). By analogy, sib‐GWAS provides evidence for PGS as a cause of sibling differences in outcomes: as she explains, *“if X [the particular value of the PGS] versus Not‐X is randomly assigned, then observing differences in outcomes that are probabilistically associated with X versus Not‐X is satisfactory evidence that X is a ‘thin’ cause of those outcomes”* [p. 109]. Under the assumptions of no interaction between a child's PGS and parental behavior, these family studies further allow for unbiased estimation of direct genetic effects, that is, the effects of the alleles inherited by a child on their own phenotype (Wolf et al., 1998; Young et al., 2018, 2019). In practice, however, it is hard to enroll family members, so family GWAS remain small and few in number compared to standard GWAS.

Moreover, the book's central focus is not on differences between family members but rather on unequal outcomes among unrelated people in the population. This is where its analogy of a genetic lottery breaks down (see also Fletcher, 2022). Biological fathers and mothers do not pair at random: people choose their partners based on geographical proximity and numerous other criteria (including family background, income, or education). Children are not raised in randomly assigned environments but often by their parents in an environment and geographic setting similar to that of their ancestors. Indeed, in many cultures, people marry and have children with particular partners precisely in the hope of avoiding the randomness of life outcomes and of improving the social prospects of their families. The fact that genetic differences play a role in generating inter‐individual differences in outcomes makes it a lottery in the Rawlsian sense of being unearned and unchosen, but not in the sense of genotypes being randomly assigned across environments. Critically, then, most of our knowledge about differences among individuals comes not from family GWAS but from standard GWAS of “unrelated” individuals, i.e., from the study of individuals from different nuclear families, whose genetic backgrounds and environmental differences are only controlled statistically (Vilhjálmsson & Nordborg, 2013).

Although Harden notes that only within‐family differences can truly be viewed as the result of a Mendelian lottery, the presentation often slips seamlessly between these two very different contexts. This elision matters: first, because it undermines the validity of the genetic lottery of meiosis as a lens through which to view genetic causes of interindividual differences; and second, because it leaps across a gulf in our understanding of GWAS findings. Genetic effects estimated in a standard GWAS include not only direct genetic effects of an individual's genotype on their phenotype, but also indirect genetic effects of the parents (and potentially siblings and peers), as well as effects of assortative mating of the parents. That is not all: because no statistical control for population structure is perfect, or even all that well defined, GWAS estimates may also include residual effects of the genetic background, and, perhaps most difficult to disentangle in humans, environmental effects that are correlated with the genetic background. Thus, PGS for traits based on standard GWAS are not estimates of direct genetic effects alone, and their predictive power derives from all these effects combined. For educational attainment, the distinction between standard GWAS and family GWAS is particularly pronounced: direct genetic effects for educational attainment are estimated to account for as little as one fourth of the variance in the PGS of a standard GWAS (Kong et al., 2020; Young et al., 2020). The remaining three‐fourths reflect a tangled mess—a braid, as Harden refers to it—of genetic and environmental effects.

Because the author is attentive to this problem, the book refers to validation studies in which the PGS from a standard GWAS is shown to be a significant predictor of within‐family differences, usually between siblings. These validations are important, because they demonstrate that the PGS's predictive power is not entirely due to indirect effects or population stratification, establishing that there is some causal genetic contribution. But given the presentation, the reader is often left to assume that the sib‐analysis fully replicates the population PGS results. For example, when reporting that the PGS for educational attainment and wealth are correlated in people of European ancestry, Harden argues for there being a causal effect based on studies showing the sibling who *“won the genetic lottery”* of a higher educational attainment PGS to be on average wealthier at retirement. She concludes that some people *“won the jump ball of genetic luck–and winning pays”* [pp. 42–43]. In fact, all the validation establishes is that when considering siblings, at least some of the correlation between the educational attainment PGS and wealth is causal; to what extent that is reflected in the differences in wealth across people from different families is far from clear.

This example points to a key conceptual difficulty with the reliance on family GWAS to explain interindividual differences: whether the reasons for sibling differences in educational attainment are even the same as those that lead to observed differences among individuals and families (Rose, 1985). As a hypothetical example, imagine that in a sib‐GWAS, variants that increase exercise are protective against coronary artery disease (CAD). From that finding, we learn that they are one cause of differences in CAD across unrelated individuals. But it does not follow that observed differences in CAD across unrelated individuals can be explained by these variants. It may be, for instance, that families who exercise more tend to consume more alcohol, or engage in other activities that put them at greater risk for CAD, counter‐balancing the effects of exercise. Every phenotype results from many causes, genetic and environmental, acting in similar or opposite directions. Consequently, differences in CAD risk in the population may have distinct explanations than those seen among siblings. This complication seems particularly salient for educational attainment, where siblings, for all their differences, are at least playing by similar rules, in contrast to people growing up in disparate educational contexts. Evidence that individual differences may have distinct sources to sibling differences comes from consideration of family studies, in which the genetic correlations between the PGS for educational attainment and other traits (e.g., BMI) disappear or are greatly reduced (Brumpton et al., 2020; Selzam et al., 2019).

That a phenotype is a convolution of many causes has important practical consequences: it makes it difficult to interpret *why* a PGS predicts interindividual outcomes. Indeed, even if a PGS is estimated from a family‐GWAS and built up entirely of direct genetic effects, once it is used to predict differences among individuals in *different* families, its predictive power can be amplified or diminished by indirect effects and population stratification, depending on whether they are correlated or anti‐correlated with the direct genetic effects.

The distinctions between family‐GWAS and standard GWAS and predictions within versus between families are therefore critical. In moving back and forth between them, the book leaves the reader confused as to the interpretation of various findings. As an illustration, after a discussion of the potential importance of parental effects, Harden talks about her own work with co‐authors (Harden et al., 2020), in which they examined *“the flow of students through the high school math curriculum as a function of their genes”* [p. 147]. In the original paper, the authors clarify that these effects need not reside in the children's “genes”: as an illustration, that parents who themselves have a high PGS for educational attainment could have more knowledge of how to navigate the school system. But neither parental effects nor the possible confounding influences of wealth are mentioned in the discussion of this example in the book. Instead, this section concludes *“Colleges and universities cannot see a student's DNA when he and she applies to college …[but] …[t]he ways in which institutions assign students, promote students, and admit students transmute invisible DNA into visible academic credentials”* [p. 148].

Moreover, where Harden notes differences between standard GWAS and sib differences, she attributes them to effects of the parents, when all we actually know is that they reflect differences among families, some of which may have accrued over generations. Below the scale of a country, there is a fine‐scale population‐genetic structure shaped by historical events played out over many hundreds of years (Leslie et al., 2015; Han et al., 2017). PGS for educational attainment reflect that structure more than most traits (Haworth et al., 2019). In other words, people are not randomized across geography; instead, there are long‐running intergenerational patterns to social mobility, with many families effectively trapped in geographic areas of greater social deprivation (Longley et al., 2021), and the ability to migrate in part influenced by heritable phenotypes (e.g., health status, Brimblecombe et al., 2000). These considerations highlight a central challenge to identifying genetic causes of behavioral traits, the immense difficulty of disentangling population stratification from biological and social effects.

## THE INTERPRETATION OF GWAS HITS FOR EDUCATIONAL ATTAINMENT AS “BUILT IN” DIFFERENCES AMONG INDIVIDUALS

In an influential thought experiment in behavioral genetics, described in *The Genetic Lottery*, Jencks imagined a society in which red‐haired children are discriminated against and not allowed to attend school (Jenckset al., 1972). In such a world, red‐haired siblings would have low educational attainment, and the loci that influence red hair would be genetic causes of low educational attainment. Yet in this example, we do not think of red hair as a cause of educational attainment in the way, as say, curiosity might be, in part because we can readily imagine a world in which the causal chain is broken. So how do we know that the PGS for educational attainment does not include manifestations of the red hair effect, i.e., whether the causes of variation in educational attainment identified in GWAS are in some sense “built in,” even if they emerge in a specific social setting, rather than mostly contingent on the society in which the individual finds themselves?

To address this question, Harden relies on the observation that significantly more of the genes near loci identified in a standard GWAS are expressed in the brain, and specifically in neurons, than expected by chance (Lee et al., 2018). In other words, there is an enrichment of associations near genes that are expressed in the brain, among other tissues. While a useful sanity check that GWAS results are not entirely spurious, this observation alone clears a low bar for a behavioral trait. Yet the book goes beyond the available evidence to conclude: *“Whatever genes are doing to make it more or less likely that some people succeed in education, they are doing it in people's brains, not their hair or livers or skin or bones”* [p. 137]. In doing so, it misrepresents the findings. There are presumably many distinct processes that contribute to educational attainment; in principle, these could include factors such as general health or childhood nutrition, which involve not only the brain but also a number of other tissues. The outcome of an enrichment analysis of an educational attainment GWAS would be, as observed, a highly statistically significant enrichment in the tissue that sits in the middle of the Venn diagram of these many different processes: the brain. Therefore, the observed enrichment cannot be taken as evidence that *all* GWAS hits act via mechanisms in the brain. Nor can the enrichment be attributed in its entirety to the GWAS individual, given that it is based on a standard GWAS and not a family one, and therefore absorbs a number of effects beyond direct genetic effects.

Furthermore, because the GWAS for any complex behavior is expected to show an enrichment in the brain, so will behavioral analogs of the red‐hair effects. Consider left handedness, for example, a trait that was discriminated against in education for generations, and for which a tissue‐enrichment analysis of GWAS hits implicates many tissues in the brain (Cuellar‐Partida et al., 2021). Similarly, it seems quite plausible that sensitivity to environmental pollutants such as lead, to which individuals are exposed very unequally, could be mediated by developmental processes playing out in the brain.

As Harden writes elsewhere, it is all too *“easy to jump to the conclusion that genetic causes must have entirely biological mechanisms, happening inside the skin”* [p. 131]. Yet reading the discussion of how genetic effects unfurl [pp. 136–137], it is hard not to interpret the book as saying that most of these GWAS effects stem from natural causes residing inside the brain, thereby nudging the reader toward genetic determinism. In the end, all we actually have, at present at least, is a large number of genetic associations, individually of tiny effect, and a statistical enrichment for a tissue that makes sense for a behavior, which is not surprising. As Harden notes, it is a very long causal chain from genetic variation to variation in educational attainment. But here, as in the elision of family and standard GWAS, we should not pretend that there are shortcuts.

## THE RELIANCE ON TYPOLOGICAL NOTIONS OF POPULATIONS

Throughout the book, Harden argues that existing PGS are a critical tool in understanding the causes of life outcome differences across individuals of European ancestry. But she is also keenly aware of the long history of racist hereditarians relying on genetic determinism to make comparisons across groups and anxious to defuse fears about how PGS may be misused in similar ways. Therefore, the book repeatedly seeks to reassure the reader that PGS do not permit group comparisons, stating *“from a statistical perspective, assuming that correlations within a group tell you something about the causes of between‐group differences is a leap that only fools would make. It is an ecological fallacy.”* [p. 86].

To date, much of the discussion in human genetics about using PGS across ancestry groups has focused on the methodological limitations of existing PGS, which are based predominantly on studies of individuals of recent European ancestry (Martin et al., 2019; Mills & Rahal, 2020). Since ancestry groups differ in linkage disequilibrium patterns and allele frequencies, these factors alone lead PGS to be increasingly poor predictors of phenotypic differences in more distantly related ancestries, and they are compounded by differences in environmental effects and gene‐by‐environment interactions (Harpak & Przeworski, 2021; Martin et al., 2019; Mostafavi et al., 2020; Privé et al., 2022; Wang et al., 2020; Yair & Coop, 2021). As Harden points out, however, these are, at least in principle, surmountable difficulties, such that she *“anticipate[s] a future in which scientists will have developed a polygenic score that is as strongly related, statistically, to academic achievement in Black students as it is in White Students”* [p. 191].

The core of her argument against group comparisons is not about the limitations of existing PGS, but about their use to study causes of phenotypic differences. There, she argues, is where the “ecological fallacy” applies: because group differences need not, and often do not, have the same causes as individual differences, *“[w]e can't “compare” the genetics of different ancestry groups using their polygenic indices”* [p. 86]. According to the argument of the book, then, GWAS is informative about causes, but only within ancestry groups.

This typological view of ancestry groups is deceiving, however. In reality, there is no bright line demarcating comparisons “within” versus “between” ancestries: there is a giant family tree of humanity, and people who share more ancestral paths through it than others, and more similar environments than others (Coop, 2017). As Barton et al. (2019) write: *“Natural populations are never homogeneous, and it is therefore misleading to imply there is a qualitative difference between ‘within‐population’ and ‘between‐population’ comparisons … With respect to confounding by population structure, the key qualitative difference is between controlling the environment experimentally, and not doing so.”* Since human groups are never compared in an experimental setting or in randomized environments, nothing ensures that environmental effects are the same across groups; for environmental factors plausibly relevant to educational attainment, they clearly are not.

The limits imposed by not controlling environmental effects also apply to comparisons among individuals within the study group, not just between groups—hence the methodological problem of population and environmental confounding in GWAS (Lander & Schork, 1994). In the end, the key difficulty is not whether comparisons are made within versus between hypothetical populations; it is all the confounding factors that exist in the absence of the ability to do experiments and how well we can measure and control for them. That is the ultimate point of Lewontin's (1970) thought experiment about corn growth mentioned in the book: not simply that *“differences between groups (such as racial groups) might be entirely caused by environmental factors, even when differences within groups are caused by genetic differences”* [p. 159], but rather that, unless the environment is carefully controlled, comparisons across individuals—even within a group—do not allow genetic causes of trait variation to be isolated (Lewontin, 1974). In that regard, the big jump taken in a GWAS is from comparisons of close family members, where the genotypes at a locus can be seen as randomized across shared environments, to comparisons of individuals from different families, where they cannot. Once that step has been taken, the tangled mess of luck, lottery, and legacy is introduced back into the study. As Harden herself notes about the ecological fallacy, it is *“a statistical point that applies anytime we are trying to jump from one level of aggregation to another”* [p. 86].

This lack of controlled environment confounds comparisons between sets of individuals in distinct settings or of differing ancestries—acutely so for individuals from different racial groups in the United States, who have been subjected to inequitable environments for generations. But it also poses a challenge *within* a racial or ethnic group. In particular, individuals who identify as Black Americans vary greatly in their proportions of recent African and European ancestries, as a consequence of the historical legacy of slavery, including the one‐drop rule and Jim Crow laws. This African ancestry traces back to disparate geographical locations in Africa, shaped by the routes of the trans‐Atlantic slave trade and migration patterns since (Micheletti et al., 2020). Within the United States, it is correlated with geography and tied to socioeconomic outcomes; for example, people with lighter skin pigmentation and lower proportion of African ancestry were more likely to leave the South during the first wave of the Great Migration (1910–1940) in search of better economic and social opportunities (Baharian et al., 2016). A GWAS within Black Americans of varying ancestry would aggregate these genetic and nongenetic effects, with only statistical controls to try to tease them apart. Since sections of the book narrate this history and emphasize the distinction between race and genetic ancestry, it is perplexing to see claims that appear to equate the two, as when Harden writes that genomics must *“become more global”* [p. 191] to develop a reliable PGS for educational attainment in Black students in the United States, or that the development of such a PGS will be an indispensable tool in distinguishing genetic from *“specific environmental causes of important developmental outcomes”* [p. 192].

In our view, these instances reflect a more general tension in the book, which arises from trying to have it both ways: to argue that PGS for educational attainment provide interpretable and meaningful predictions of inter‐individual differences that reflect underlying genetic causes, yet to claim that they have no validity beyond hypothetical ancestry group boundaries. As we have laid out, we believe instead that current PGS for educational attainment are neither interpretable nor particularly meaningful. GWAS undoubtedly captures some causal genetic effects, that is, more than confounding alone, and there is interesting science to learn from these initial findings. But we currently understand next to nothing about the causal paths from GWAS findings to educational attainment, notably the extent to which they include analogs of Jencks‐style “red hair effects” and the legacy of accumulated indirect effects. That may not matter when PGS for EA are to be employed as a statistical tool in the study of the impact of local social interventions, but it matters greatly when they are used to elucidate, let alone redress, social inequalities.

Given these limitations, we do not see what the field of genetics has to add to the conversation about redistributive justice, beyond confirming what has long been recognized—that life outcomes differ for all kinds of reasons beyond people's control—and we very much doubt that overstating our understanding of the genetics of behaviors is going to increase empathy. As the author appreciates, there is a history of this kind of practice, invariably with nefarious consequences, yet every generation seems to believe that their technological twist will help them to avoid the same pitfalls. Despite its engaging narrative, *The Genetic Lottery* therefore leaves us unconvinced, and with the impression of genomics serving as a distraction from much more exigent political conversations (e.g., National Academies of Sciences, Engineering, and Medicine, 2019).

## References

[evo14449-bib-0001] Baharian, S. , Barakatt, M. , Gignoux, C.R. , Shringarpure, S. , Errington, J. , Blot, W.J. , Bustamante, C.D. , Kenny, E.E. , Williams, S.M. , Aldrich, M.C. , et al. (2016) The great migration and African‐American genomic diversity. PLoS Genetics, 12(5), e1006059. 27232753 10.1371/journal.pgen.1006059 PMC4883799

[evo14449-bib-0002] Barcellos, S.H. , Carvalho, L.S. & Turley, P. (2018) Education can reduce health differences related to genetic risk of obesity. Proceedings of the National Academy of Sciences of the United States of America, 115(42), E9765–E9772. 30279179 10.1073/pnas.1802909115 PMC6196527

[evo14449-bib-0003] Barton, N. , Hermisson, J. & Nordborg, M. (2019) Population genetics: Why structure matters. Elife, 8, e45380. 30895925 10.7554/eLife.45380 PMC6428565

[evo14449-bib-0004] Barton, N.H. & Keightley, P.D. (2002) Understanding quantitative genetic variation. Nature Reviews Genetics, 3(1), 11–21. 10.1038/nrg700 11823787

[evo14449-bib-0005] Branigan, A.R. , McCallum, K.J. & Freese, J. (2013) Variation in the heritability of educational attainment: An international meta‐analysis. Social Forces, 92(1), 109–140.

[evo14449-bib-0006] Brimblecombe, N. , Dorling, D. & Shaw, M. (2000) Migration and geographical inequalities in health in Britain. Social Science & Medicine, 50(6), 861–878. 10695983 10.1016/s0277-9536(99)00371-8

[evo14449-bib-0007] Brumpton, B. , Sanderson, E. , Heilbron, K. , Hartwig, F.P. , Harrison, S. , Vie, G.Å. , Cho, Y. , Howe, L.D. , Hughes, A. , Boomsma, D.I. , et al. (2020) Avoiding dynastic, assortative mating, and population stratification biases in mendelian randomization through within‐family analyses. Nature Communications, 11(1), 1–13. 10.1038/s41467-020-17117-4 PMC7360778 32665587

[evo14449-bib-0008] Coop, G. (2017) Where did your genetic ancestors come from? https://gcbias.org/2017/12/19/1628/.

[evo14449-bib-0009] Cuellar‐Partida, G. , Tung, J.Y. , Eriksson, N. , Albrecht, E. , Aliev, F. , Andreassen, O.A. , Barroso, I. , Beckmann, J.S. , Boks, M.P. , Boomsma, D.I. , et al. (2021) Genome‐wide association study identifies 48 common genetic variants associated with handedness. Nature Human Behaviour, 5(1), 59–70. 10.1038/s41562-020-00956-y PMC7116623 32989287

[evo14449-bib-0010] Downes, S.M. & Turkheimer, E. (2021) An early history of the heritability coefficient applied to humans (1918–1960). Biological Theory, 1–12.

[evo14449-bib-0011] Feldman, M.W. & Lewontin, R.C. (1975) The Heritability Hang‐up. Science, 190(4220), 1163–1168. 1198102 10.1126/science.1198102

[evo14449-bib-0012] Fletcher, J.M. (2022) Backdoor to a Dead End: A Review Essay. Population and Development Review. In Press.

[evo14449-bib-0013] Han, E. , Carbonetto, P. , Curtis, R.E. , Wang, Y. , Granka, J.M. , Byrnes, J. , Noto, K. , Kermany, A.R. , Myres, N.M. , Barber, M.J. , Rand, K.A. , Song, S. , Roman, T. , Battat, E. , Elyashiv, E. , Guturu, H. , Hong, E.L. , Chahine, K.G. , & Ball, C.A. (2017) Clustering of 770,000 genomes reveals post‐colonial population structure of North America. Nature Communications, 8, 14238. 10.1038/ncomms14238 PMC5309710 28169989

[evo14449-bib-0014] Harden, K.P. (2021) The genetic lottery: why DNA matters for social equality. Princeton University Press.

[evo14449-bib-0015] Harden, K.P. , Domingue, B.W. , Belsky, D.W. , Boardman, J.D. , Crosnoe, R. , Malanchini, M. , Nivard, M. , Tucker‐Drob, E.M. & Harris, K.M. (2020) Genetic associations with mathematics tracking and persistence in secondary school. NPJ Science of Learning, 5(1), 1–8. 32047651 10.1038/s41539-020-0060-2 PMC7002519

[evo14449-bib-0016] Harpak, A. & Przeworski, M. (2021) The evolution of group differences in changing environments. PLoS Biology, 19(1), e3001072. 33493148 10.1371/journal.pbio.3001072 PMC7861633

[evo14449-bib-0017] Haworth, S. , Mitchell, R. , Corbin, L. , Wade, K.H. , Dudding, T. , Budu‐Aggrey, A. , Carslake, D. , Hemani, G. , Paternoster, L. , Smith, G.D. , et al. (2019) Apparent latent structure within the UK biobank sample has implications for epidemiological analysis. Nature Communications, 10(1), 1–9. 10.1038/s41467-018-08219-1 PMC6338768 30659178

[evo14449-bib-0018] Jencks, C. et al. (1972) Inequality: A reassessment of the effect of family and schooling in America. New York: Basic Books.

[evo14449-bib-0019] Kemper, K.E. , Yengo, L. , Zheng, Z. , Abdellaoui, A. , Keller, M.C. , Goddard, M.E. , Wray, N.R. , Yang, J. & Visscher, P.M. (2021) Phenotypic covariance across the entire spectrum of relatedness for 86 billion pairs of individuals. Nature Communications, 12(1), 1–11. 10.1038/s41467-021-21283-4 PMC7886899 33594080

[evo14449-bib-0020] Kong, A. , Benonisdottir, S. & Young, A.I. (2020) Family analysis with mendelian imputations. BioRxiv.

[evo14449-bib-0021] Kong, A. , Thorleifsson, G. , Frigge, M.L. , Vilhjalmsson, B.J. , Young, A.I. , Thorgeirsson, T.E. , Benonisdottir, S. , Oddsson, A. , Halldorsson, B.V. , Masson, G. , et al. (2018) The nature of nurture: Effects of parental genotypes. Science, 359(6374), 424–428. 29371463 10.1126/science.aan6877

[evo14449-bib-0022] Lander, E.S. & Schork, N.J. (1994) Genetic dissection of complex traits. Science, 265(5181), 2037–2048. 8091226 10.1126/science.8091226

[evo14449-bib-0023] Lee, J.J. , Wedow, R. , Okbay, A. , Kong, E. , Maghzian, O. , Zacher, M. , Nguyen‐Viet, T.A. , Bowers, P. , Sidorenko, J. , Linnér, R.K. , et al. (2018) Gene discovery and polygenic prediction from a 1.1‐million‐person gwas of educational attainment. Nature Genetics, 50(8), 1112. 30038396 10.1038/s41588-018-0147-3 PMC6393768

[evo14449-bib-0024] Leslie, S. , Winney, B. , Hellenthal, G. , Davison, D. , Boumertit, A. , Day, T. , Hutnik, K. , Royrvik, E.C. , Cunliffe, B. , Lawson, D.J. , et al. (2015) The fine‐scale genetic structure of the british population. Nature, 519(7543), 309–314. 25788095 10.1038/nature14230 PMC4632200

[evo14449-bib-0025] Lewontin, R.C. (1970) Race and intelligence. Bulletin of the Atomic Scientists, 26(3), 2–8.

[evo14449-bib-0026] Lewontin, R.C. (1974) The analysis of variance and the analysis of causes. American Journal of Human Genetics, 26, 400–411. 4827368 PMC1762622

[evo14449-bib-0027] Longley, P.A. , van Dijk, J. & Lan, T. (2021) The geography of intergenerational social mobility in Britain. Nature Communications, 12(1), 6050. 10.1038/s41467-021-26185-z PMC8548290 34702809

[evo14449-bib-0028] Martin, A.R. , Kanai, M. , Kamatani, Y. , Okada, Y. , Neale, B.M. & Daly, M.J. (2019) Clinical use of current polygenic risk scores may exacerbate health disparities. Nature Genetics, 51(4), 584–591. 30926966 10.1038/s41588-019-0379-x PMC6563838

[evo14449-bib-0029] Martschenko, D.O. (2021) Social equality in an alternate world. Hastings Center Report, 51(6), 54–55. 34904740 10.1002/hast.1307 PMC9210985

[evo14449-bib-0030] Micheletti, S.J. , Bryc, K. , Esselmann, S.G.A. , Freyman, W.A. , Moreno, M.E. , Poznik, G.D. , Shastri, A.J. , Agee, M. , Aslibekyan, S. , Auton, A. , et al. (2020) Genetic consequences of the transatlantic slave trade in the americas. The American Journal of Human Genetics, 107(2), 265–277. 32707084 10.1016/j.ajhg.2020.06.012 PMC7413858

[evo14449-bib-0031] Mills, M.C. & Rahal, C. (2020) The GWAS diversity monitor tracks diversity by disease in real time. Nature Genetics, 52(3), 242–243. 32139905 10.1038/s41588-020-0580-y

[evo14449-bib-0032] Morrissey, M.B. , Kruuk, L.E. & Wilson, A.J. (2010) The danger of applying the breeder's equation in observational studies of natural populations. Journal of Evolutionary Biology, 23(11), 2277–2288. 20831731 10.1111/j.1420-9101.2010.02084.x

[evo14449-bib-0033] Mostafavi, H. , Harpak, A. , Agarwal, I. , Conley, D. , Pritchard, J.K. & Przeworski, M. (2020) Variable prediction accuracy of polygenic scores within an ancestry group. eLife, 9, e48376. 31999256 10.7554/eLife.48376 PMC7067566

[evo14449-bib-0034] National Academies of Sciences, Engineering, and Medicine . (2019) A Roadmap to Reducing Child Poverty. Washington, DC: The National Academies Press. 31593382

[evo14449-bib-0035] Panofsky, A. (2021) Biology meets public policy. Science, 373(6562), 1449–1449.

[evo14449-bib-0036] Parens, E. (2021) Will sociogenomics reduce social inequality? https://www.thehastingscenter.org/will‐sociogenomics‐reduce‐social‐inequality/.

[evo14449-bib-0037] Price, A.L. , Zaitlen, N.A. , Reich, D. & Patterson, N. (2010) New approaches to population stratification in genome‐wide association studies. Nature Reviews Genetics, 11(7), 459–463. 10.1038/nrg2813 PMC2975875 20548291

[evo14449-bib-0038] Privé, F. , Aschard, H. , Carmi, S. , Folkersen, L. , Hoggart, C. , O'Reilly, P.F. & Vilhjálmsson, B.J. (2022) Portability of 245 polygenic scores when derived from the UK biobank and applied to 9 ancestry groups from the same cohort. The American Journal of Human Genetics, 109(1), 12–23. 34995502 10.1016/j.ajhg.2021.11.008 PMC8764121

[evo14449-bib-0039] Rawls, J. (1971) A Theory of Justice (1 ed.). Cambridge, Massachussets: Belknap Press of Harvard University Press.

[evo14449-bib-0040] Rose, G. (1985) Sick individuals and sick populations. International Journal of Epidemiology, 14(1). 10.1093/ije/14.1.32 3872850

[evo14449-bib-0041] Selzam, S. , Ritchie, S.J. , Pingault, J.‐B. , Reynolds, C.A. , O'Reilly, P.F. & Plomin, R. (2019) Comparing within‐and between‐family polygenic score prediction. The American Journal of Human Genetics, 105(2), 351–363. 31303263 10.1016/j.ajhg.2019.06.006 PMC6698881

[evo14449-bib-0042] Tabery, J. (2008) RA Fisher, Lancelot Hogben, and the origin(s) of genotype–environment interaction. Journal of the History of Biology, 41(4), 717–761. 19244846 10.1007/s10739-008-9155-y

[evo14449-bib-0043] Turkheimer, E. (2000) Three laws of behavior genetics and what they mean. Current Directions in Psychological Science, 9(5), 160–164.

[evo14449-bib-0044] Vilhjálmsson, B.J. & Nordborg, M. (2013) The nature of confounding in genome‐wide association studies. Nature Reviews Genetics, 14(1), 1–2. 10.1038/nrg3382 23165185

[evo14449-bib-0045] Visscher, P.M. , Hill, W.G. & Wray, N.R. (2008) Heritability in the genomics era—concepts and misconceptions. Nature Reviews Genetics, 9(4), 255–266. 10.1038/nrg2322 18319743

[evo14449-bib-0046] Wang, Y. , Guo, J. , Ni, G. , Yang, J. , Visscher, P.M. & Yengo, L. (2020) Theoretical and empirical quantification of the accuracy of polygenic scores in ancestry divergent populations. Nature Communications, 11(1), 1–9. 10.1038/s41467-020-17719-y PMC7395791 32737319

[evo14449-bib-0047] Wolf, J.B. , Brodie III, E.D. , Cheverud, J.M. , Moore, A.J. & Wade, M.J. (1998) Evolutionary consequences of indirect genetic effects. Trends in Ecology & Evolution, 13(2), 64–69. 21238202 10.1016/s0169-5347(97)01233-0

[evo14449-bib-0048] Wray, N.R. , Kemper, K.E. , Hayes, B.J. , Goddard, M.E. & Visscher, P.M. (2019) Complex trait prediction from genome data: contrasting ebv in livestock to prs in humans: genomic prediction. Genetics, 211(4), 1131–1141. 30967442 10.1534/genetics.119.301859 PMC6456317

[evo14449-bib-0049] Yair, S. & Coop, G. (2021) Population differentiation of polygenic score predictions under stabilizing selection. bioRxiv. 10.1098/rstb.2020.0416 PMC9014188 35430887

[evo14449-bib-0050] Young, A.I. , Benonisdottir, S. , Przeworski, M. & Kong, A. (2019) Deconstructing the sources of genotype‐phenotype associations in humans. Science, 365(6460), 1396–1400. 31604265 10.1126/science.aax3710 PMC6894903

[evo14449-bib-0051] Young, A.I. , Frigge, M.L. , Gudbjartsson, D.F. , Thorleifsson, G. , Bjornsdottir, G. , Sulem, P. , Masson, G. , Thorsteinsdottir, U. , Stefansson, K. & Kong, A. (2018) Relatedness disequilibrium regression estimates heritability without environmental bias. Nature Genetics, 50(9), 1304–1310. 30104764 10.1038/s41588-018-0178-9 PMC6130754

[evo14449-bib-0052] Young, A.I. , Nehzati, S.M. , Lee, C. , Benonisdottir, S. , Cesarini, D. , Benjamin, D.J. , Turley, P. & Kong, A. (2020) Mendelian imputation of parental genotypes for genome‐wide estimation of direct and indirect genetic effects. BioRxiv. 10.1038/s41588-022-01085-0 PMC9197765 35681053

